# Early-Age Cracking Behavior of Concrete Slabs with GFRP Reinforcement

**DOI:** 10.3390/ma16155489

**Published:** 2023-08-06

**Authors:** Hossein Roghani, Antonio Nanni, John E. Bolander

**Affiliations:** 1 Department of Civil and Architectural Engineering, University of Miami, Coral Gables, FL 33146, USA; h.roghani@miami.edu (H.R.); nanni@miami.edu (A.N.); 2 Department of Civil and Environmental Engineering, University of California, Davis, CA 95616, USA

**Keywords:** concrete, GFRP reinforcement, early-age cracking, plastic shrinkage, lattice modeling

## Abstract

This paper reports on a combined experimental and numerical modeling investigation of cracking of concrete slabs with GFRP reinforcement. At this stage of the project, attention is given to early-age cracking driven by plastic shrinkage, preceding longer term considerations of cracking resistance over the service life of field applications. Of interest is the effectiveness of GFRP reinforcement in restricting plastic shrinkage cracking. Nine small-scale slab specimens were subjected to controlled evaporation rates. Images of crack development were acquired periodically, from which crack width estimations were made. Comparisons were made between slabs reinforced with conventional steel and those reinforced with GFRP, along with control specimens lacking reinforcement. During the period of plastic shrinkage, the time of crack initiation and subsequent crack openings do not appear to be influenced by the presence of the reinforcing bars. To understand this behavior, six early-age bond tests were conducted for both types of the bars after 1, 2, and 3 h exposure to the controlled evaporation rate. In addition, concrete strength development and time of settings were measured using penetration resistance tests on a representative mortar. The numerical modeling component of this research is based on a Voronoi cell lattice model; in this approach, the relative humidity, temperature, and displacement fields are discretized in three-dimensions, allowing for a comprehensive investigation of material behavior within the controlled environment. Based on the measured bond properties, our simulations confirm that the reinforcing bars restrict crack development, though they do not prevent it entirely.

## 1. Introduction

Corrosion of the reinforcing steel is a primary factor affecting the durability of structural concrete, especially during exposure to severe environments. The potential for corrosion-induced durability problems can be reduced by controlling crack formation in the concrete, particularly at early ages when the concrete is susceptible to cracking. Alternatively, non-corroding materials can be used as concrete reinforcement.

Plastic cracking is one of the earliest faults in concrete elements and, if not addressed, can reduce the durability and service life of the structure. Cracks act as pathways for detrimental chemicals and other species that can foster corrosion of embedded steel reinforcement [1,2]. Many factors affect plastic cracking of concrete. Evaporation of water, bleeding of water, settlement of solid particles within the concrete mixture, capillary action, and surface finishing have been identified as the primary mechanisms responsible for plastic cracking of concrete [3]. Plastic shrinkage cracking takes place when the water evaporation rate exceeds the rate at which bleed water is supplied to the drying surface [4]. Uno [5] confirmed that evaporation of water, fine aggregate content, water to cement ratio, admixtures, member size, and construction practices are influential parameters affecting plastic shrinkage cracking. In hot, dry, and windy regions the freshly placed concrete is vulnerable to plastic shrinkage cracking. These cracks frequently appear on the surfaces of flat structural elements such as pavements, flat slabs, bridge decks, beams, and slabs-on-ground [5,6,7,8]. Shrinkage of the concrete becomes problematic when the concrete is restrained [9,10]. Restrained shrinkage induces tensile stresses in the concrete, which can lead to cracking [11].

The present research involves the use of glass fiber-reinforced polymer (GFRP) bars to reinforce concrete exposed to severe conditions such as those presented by marine environments. GFRP rebars are employed to avoid the risk of corrosion in concrete structures exposed to harsh marine environments or de-icing salts. The durability of internal reinforcement is critical to the service life of these structures [12]. Development of FRP reinforcement for concrete structures began in the 1960s [13] and has become a matter of global interest [14,15]. By the late 1980s, FRP bars had become more widely recognized as a reasonable alternative for conventional steel reinforcement in structural concrete, particularly when the market need for electromagnetically-transparent reinforcing bars rose [12]. GFRP bars have various advantages over steel, including corrosion resistance, electromagnetic neutrality, a high strength-to-weight ratio, and a high fatigue tolerance. Furthermore, the material’s lower unit weight (one-fourth that of steel) reduces transportation and installation costs [12,16,17,18]. The bond between the FRP and concrete materials typically governs the performance of the composite system, which is a subject of continued interest [19,20,21].

Despite the fact that FRP bars have been successfully used for structural reinforcement in concrete members in building and bridge projects for the past 30 years, there has only recently been interest in using FRP bars and meshes as secondary reinforcement in place of conventional temperature and shrinkage steel reinforcement for non-structural concrete members such as plain concrete footings, concrete slabs-on-ground, and plain concrete walls [22]. Of interest is the effectiveness of such reinforcement in restricting plastic shrinkage cracking at early ages. It is known that conventional steel reinforcement does not restrict the opening of plastic shrinkage cracks. However, the nature of the bond mechanisms is potentially different, and in relative terms the mismatch in stiffness between GFRP and immature concrete is smaller.

Herein, the performance of GFRP bars relative to that of conventional steel reinforcement is investigated through a combination of physical experimentation and numerical modeling. The bases for the experiments and modeling exercises are first described, after which the results are presented. In laboratory tests, the type of reinforcement does not significantly influence the time of cracking and crack opening driven by plastic shrinkage. The modeling exercises indicate a dependence of crack opening on the type of reinforcement, and otherwise produce similar results.

## 2. Materials

### 2.1. Concrete

Various concrete mixture designs were evaluated to increase the probability of inducing plastic shrinkage cracking. The concrete was designed according to the provisions of the ACI 211.1-91 [23]. This concrete mixture does not contain any admixtures or supplementary cementitious materials and is designed with high cement content, which increases plastic shrinkage. Almusallam et al. [24] reported that higher cement content results in higher crack intensity. The concrete was made using Type I/II Portland cement and locally available coarse and fine aggregates. No. 89 small coarse aggregate was used to increase the concrete plastic shrinkage [25]. The cement composition and concrete mixture proportions are shown in Table 1 and Table 2, respectively. To increase the rate of evaporation of water from the exposed concrete surfaces, the mixing water was heated to 50 °C before adding it to the other ingredients. Increasing the mixing water temperature may cause higher bleeding and segregation of the mixture components. The 50 °C temperature was chosen to obtain desirable bleed water, slump, and compressive strength properties [26]. Both coarse and fine aggregates were fully dried before being added to the concrete. One goal of drying the aggregates is to maintain consistency in the temperature of the concrete by ensuring that the same amount of heated water is utilized in all castings and there is no water coming from moist aggregates. Furthermore, dry aggregates tend to lessen the amount of bleed water.

### 2.2. GFRP and Steel Reinforcing Bars

Three different types of slabs were considered. Along with plain concrete slabs that served as control specimens, the slabs were reinforced with either conventional steel or GFRP bars, both having a 9.5 mm (0.375 in) nominal diameter. The steel bars used in this study were conventional steel reinforcement bars with a modulus of elasticity of 200 GPa (29.0 Msi). The physical and mechanical characteristics of the GFRP bars are presented in Table 3 and Table 4.

## 3. Experimental Program

### 3.1. Plastic Shrinkage Test

**Figure 1:**
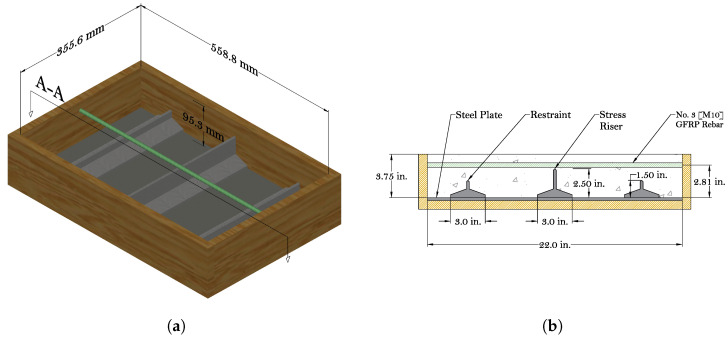
Device for simulating plastic shrinkage cracking: (**a**) 3D sketch of the device and (**b**) section A-A.

Potential for plastic shrinkage cracking was assessed using a modified form of the setup described in ASTM C1579-21 [27]. The setup involves small-scale slab specimens that incorporate metallic inserts attached to a formwork base. The insert is a 6.35 mm (0.25 in) steel plate with three welded T-bars, as illustrated in Figure 1. The T-bars at both ends restrain plastic shrinkage of the concrete, which fosters crack formation, while the one at mid-span acts as a stress riser that influences the point of crack initiation. The two long sides of the mold between the restraints were oiled to facilitate bond breaking and shrinkage; however, the short sides of the mold were not oiled in order to intensify the restraining effect at both ends. For the purpose of bond breaking, sheets of polyester film were attached to the steel baseplate between the riser and restraints. Whereas ASTM C1579-21 pertains to the testing of fiber reinforced (or plain) concrete materials, a reinforcing bar with a 19 mm (0.75 in) concrete cover was positioned within each of the reinforced specimens, as shown in Figure 1a. The applicability of the plastic shrinkage test to other bar diameters or other concrete covers needs to be addressed in another study. The concrete surface is sometimes textured to improve skid resistance or other performance objectives. The influence of such texturing on the transport of bleed water and subsequent drying of the concrete surface is another matter for additional study.

The main challenge in this testing approach is to consistently ensure the occurrence of cracking in the early stages. Therefore, an environmental chamber was designed and built to provide the critical rate of evaporation for plastic shrinkage cracking. The environmental conditions were controlled for the first 6 h. On the average, for the entire set of slab specimens, the following conditions were maintained: a wind velocity of 7.8 m/s, a relative humidity of 37%, and an ambient temperature of 32.2 °C. Based on measurements made at 30 min intervals, the average standard deviations of these quantities were 0.46 m/s, 2.60% and 1.53 °C, respectively. This combination caused plastic shrinkage cracking in all specimens. In addition, crack initiation and development were monitored by acquiring images of the drying surface. Postprocessing of the images provided data regarding the time of crack initiation and evolution of crack openings.

### 3.2. Early-Age Bond Test

**Figure 2:**
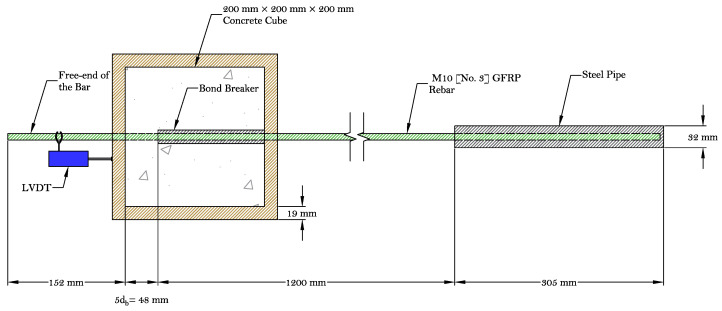
Early-age bond test specimen.

**Figure 3:**
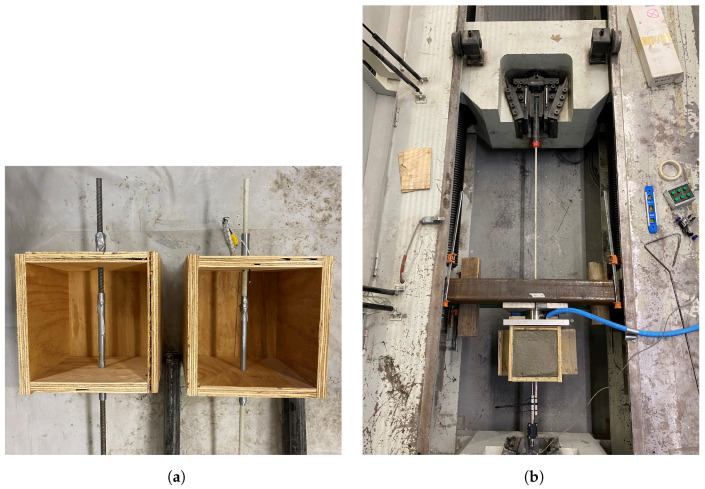
Early-age bond test: (**a**) bond test molds and (**b**) test setup.

The early-age bond behavior between the reinforcement and concrete was evaluated using ASTM D7913-14 [28]. Controlling crack formation depends critically on how well the reinforcement is bonded to the concrete. The bond specimen configuration is illustrated in Figure 2. Six bond samples were prepared according to the ASTM D7913-14: three for GFRP and three for steel bars. The samples were tested after being exposed to the prescribed conditions of the environmental chamber for 1, 2, and 3 h. Elevated curing temperature can influence the bond behavior of concrete with GFRP bars; however, in this study, the average chamber temperature was 32.2 °C, making this effect insignificant [29]. The same concrete mixture that was used for the plastic shrinkage tests was used for the bond tests. Using the same mixture design, a companion set of pullout tests according to ASTM D7913-14 was conducted to measure the bond properties of the GFRP bars at early age. A 2000 kN (450-kips) horizontal Universal Testing Machine (UTM) was used to run the pull-out tests. The displacement–control loading procedure was implemented and the load was applied with a rate of 1.3 mm/min (0.050 in/min). A 440 kN (100-kip) load cell was used to record the applied load, and a Linear Variable Differential Transformer (LVDT) was mounted at the free-end of the bar to measure free-end slip. The test setup is illustrated in Figure 3b.

### 3.3. Penetration Test

The ASTM C403-16 [30] Standard Test Method for Time of Setting of Concrete Mixtures by Penetration Resistance was conducted to measure setting times and evaluate strength development in the first several hours after casting. These data were used in the modeling approach described later in this paper. Three 150 mm × 150 mm × 150 mm (6 in × 6 in × 6 in) specimens were prepared by sieving the concrete mixture through a 4.75-mm sieve to obtain the representative mortar required by ASTM C403-16.

## 4. Experimental Results

### 4.1. Plastic Shrinkage Test

**Figure 4:**
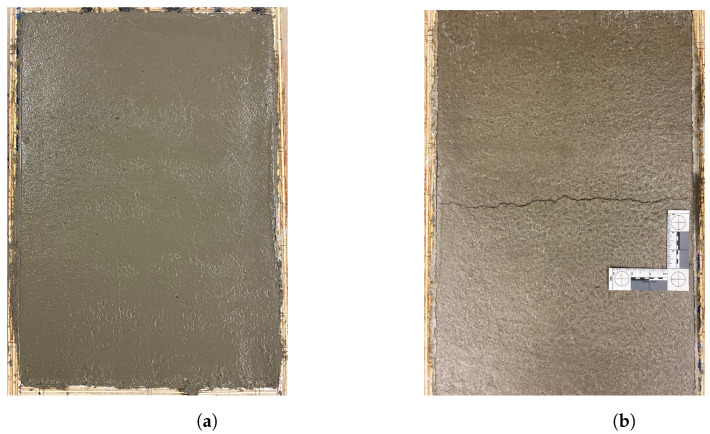
A typical plastic shrinkage specimen: (**a**) after surface finishing and (**b**) after plastic shrinkage cracking.

**Figure 5:**
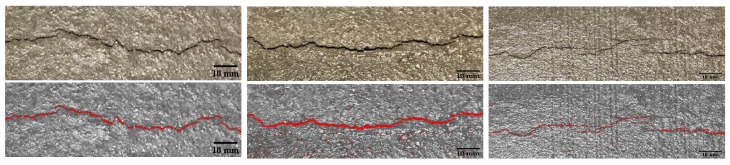
Original (**above**) and postprocessed (**below**) images of cracking of the slab surface above the stress riser; from **left** to **right**: P-SC-1, S-SC-1, G-SC-1.

Along with the time of crack onset, Table 5 provides the measured crack area, length, and calculated average crack width after six hours of exposure to the specified evaporation conditions. A typical concrete specimen after surface finishing and after cracking is illustrated in Figure 4. All the cracks appeared between 113 to 141 min after the first contact between the cement and water. Typical raw and postprocessed images of the middle portion of the cracked specimens are presented in Figure 5. Crack area and crack length were measured using the ImageJ platform for image analysis. Crack widths did not noticeably narrow in the vicinity of reinforcement, and were rather uniform over the crack lengths. The premature bond between the reinforcement and concrete during this time span results in poor stress transfer between the concrete and reinforcement. As expected, the reinforcement appears to be ineffective in controlling plastic shrinkage cracking.

**Figure 6:**
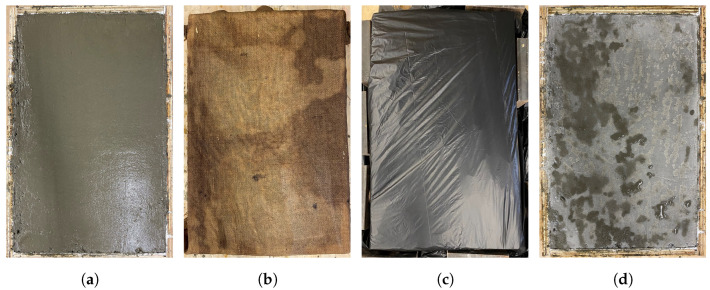
Cured specimen: (**a**) after casting, (**b**) with wet burlap, (**c**) under plastic, and (**d**) after 18 h curing.

A control specimen of plain concrete was cast and allowed to cure for 18 h in a laboratory environment. This specimen did not show any signs of cracking (see Figure 6d), which confirms that plastic shrinkage cracking can be prevented by considering an effective curing condition to supply water to the drying surface of concrete and casting in an environmental condition where the rate of evaporation of water is not critical.

**Figure 7:**
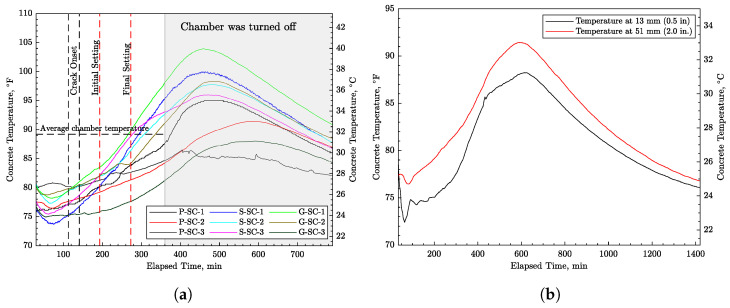
Concrete temperature development: (**a**) for all nine specimens at depth of 51 mm (2 in) and (**b**) at a different depth for specimen P-SC-2.

Figure 7a shows the temperature development in all nine concrete specimens as measured by a thermocouple device embedded at a depth of 51 mm (2 in) below the concrete surface. The average ambient temperature within the environmental chamber is indicated in the figure as well. According to Figure 7a, the concrete temperature at crack onset ranges from about 24 to 28 °C (75 to 81 °F). A temperature drop occurs shortly after casting in the majority of specimens, which, as described later, is likely due to the effects of evaporative cooling. Specimen P-SC-2 was measured with an additional thermocouple located at a depth of 13 mm (0.5 in) to gauge the significance of temperature variation over the concrete depth. As shown in Figure 7b, temperature lessens when approaching the concrete surface due to both evaporative and convective heat exchange with the environment.

**Figure 8:**
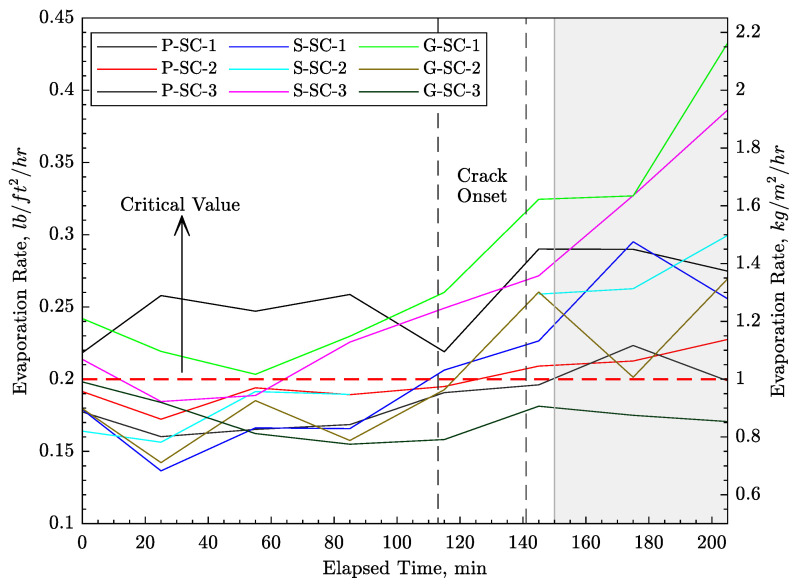
Estimated evaporation rate of water from the concrete surface.

Figure 8 presents estimates of evaporation rate for all nine specimens, which were calculated by inserting concrete temperature, wind velocity, ambient temperature, and relative humidity into Uno’s formula [5]. The graphs show that the rate of evaporation is lower in the first stages after concrete casting. This lower rate can be explained in part by the concrete’s temperature development starting roughly from the time of crack initiation. The gray area in Figure 8 shows the beginning of strength development based on the penetration resistance results as described later. The increased rate of evaporation for some specimens in this period is not of primary interest, as the concrete has already cracked.

**Figure 9:**
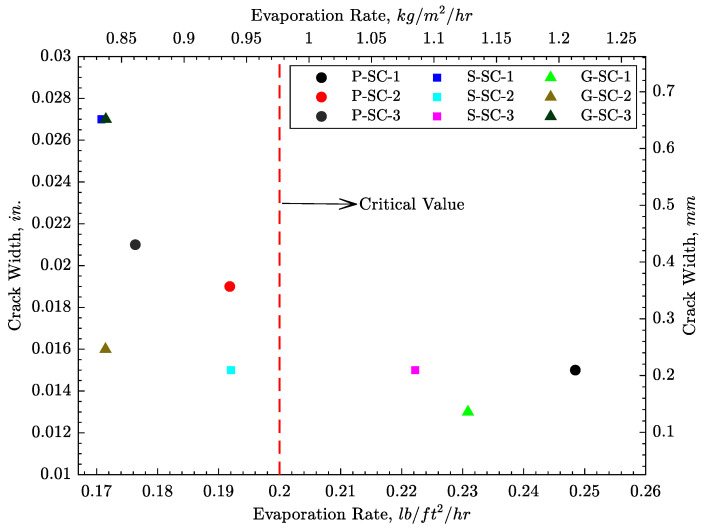
Measured crack width versus calculated evaporation rate.

In Figure 9, the measured crack widths are plotted against the calculated average evaporation rates. The average value of the evaporation rate was calculated for each measurement up to the time of crack initiation. It was observed that plastic shrinkage cracking can occur for evaporation rates that are even below the critical value of 1.0 kg/m${}^{2}$/h (0.2 lb/ft${}^{2}$/h) recommended by ACI 305R-20 [31]. This observation has been made by other researchers as well [24].

### 4.2. Early-Age Bond Test

To understand the ineffectiveness of the reinforcement in controlling plastic shrinkage cracking, as observed in plastic shrinkage testing, early-age bond tests were conducted to evaluate the bond strength of reinforcement with fresh concrete. It was found that the bond strength at *t* = 2 h (the approximate time of crack onset) is only about 0.14 MPa. For comparison, the ASTM D7957-22 [32] requires a minimum guaranteed bond strength of 7.6 MPa after 28 days of concrete hardening, where the guaranteed value is the mean minus three standard deviations of the sample strengths. It should be noted that in order to avoid disturbing the freshly poured concrete, the bond samples were tested without the formwork being removed.

**Figure 10:**
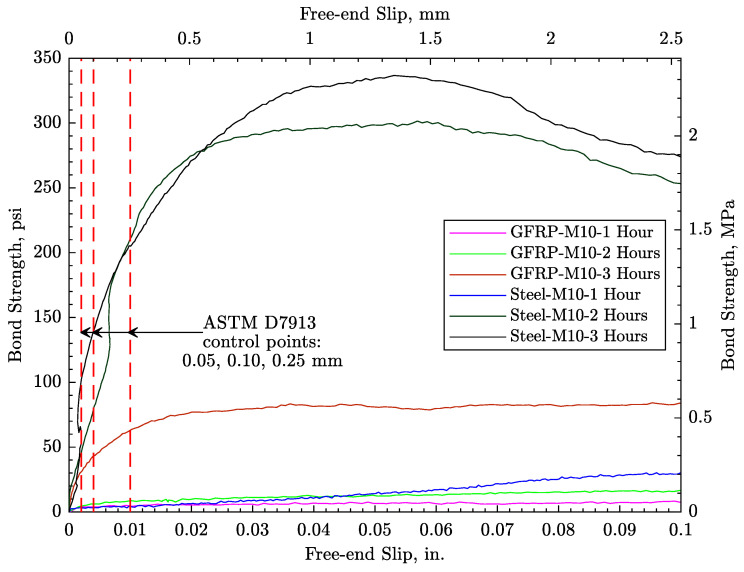
Bond stress–slip relationships.

**Figure 11:**
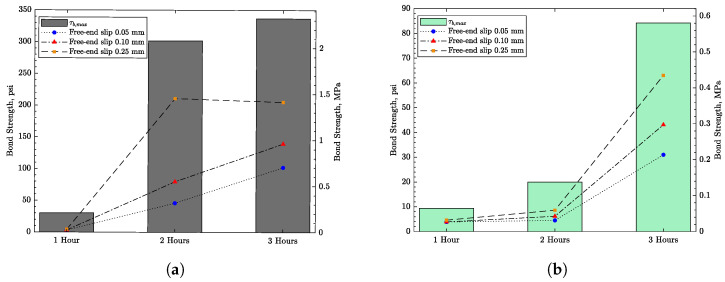
Pull-out test results: (**a**) steel reinforcement and (**b**) GFRP reinforcement.

**Figure 12:**
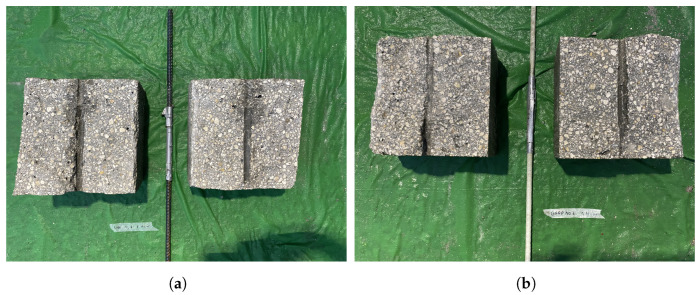
Representative bond samples after failure: (**a**) Steel No. 3 at 1 h and (**b**) GFRP No. 3 at 1 h.

Figure 10 and Figure 11 show the bond strength test results for both steel and GFRP reinforcement of the concrete. Steel reinforcement shows a higher bond strength after 1, 2, and 3 h exposure to the controlled environment. Nevertheless, both types of reinforcement show an insignificant bond strength after 1 and 2 h exposure, which is roughly the time at which that plastic shrinkage cracks occurred. In addition, a free-end slip of nearly 0.5 mm (0.02 in) is required to achieve the peak bond strength, suggesting that the observed crack widening occurs before the peak strength has been reached. To assess the bond surface between the bars and the concrete, the samples were cut in half after test completion, as shown in Figure 12 for two representative samples. The specimens appeared to fail from direct pullout of the bars, without evidence of radial cracking or other forms of damage local to the bar–concrete interface.

### 4.3. Penetration Test

**Figure 13:**
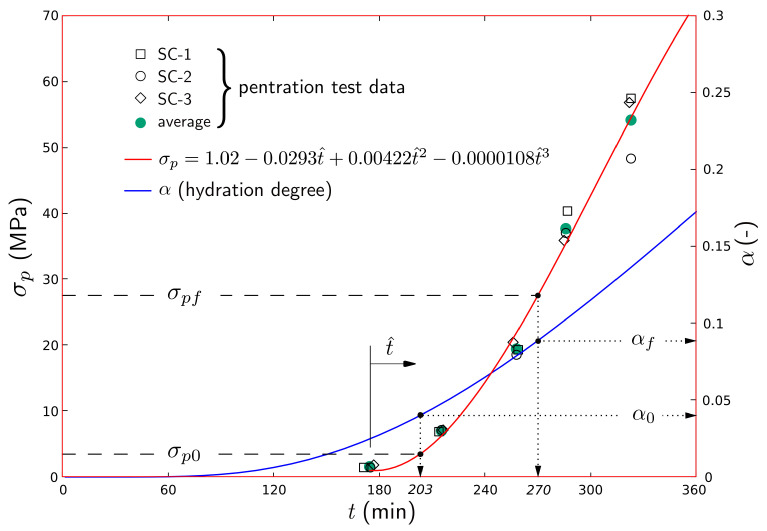
Penetration resistance, time of setting, and simulated degrees of hydration.

Figure 13 shows the concrete strength development based on penetration test data from a modified ASTM C403-16 procedure. The test deviated from the standard procedure in three respects: the use of heated water and dry aggregates for the concrete mixture, and holding of the test samples within the same controlled environment used for the plastic shrinkage testing. This was done to obtain strength measurements able to account for the conditions of the slab specimens. Table 6 shows the penetration resistance and setting times for each individual test. The average penetration resistances for each set of measurements are fitted using a cubic polynomial, as shown in Figure 13, where $\hat{t}$ measures the time from the first set of tests. The initial and final setting times obtained from the fitted curve are 203 and 270 min, respectively. In all specimens, the crack occurred before initial setting of the concrete.

## 5. Modeling Approach

Voronoi cell–lattice models (VCLM) are used to simulate the early-age thermal and hygral fields acting in the concrete as well as the mechanical response to those actions. Such discrete modeling approaches are particularly effective in simulating cracking and other forms of displacement discontinuity [33,34] while capturing relevant aspects of continuum behavior [35,36]. Lattice-based simulations have been used to model the strength and durability of concrete under various types of loading [37,38,39,40,41,42,43,44,45,46,47,48].

**Figure 14:**
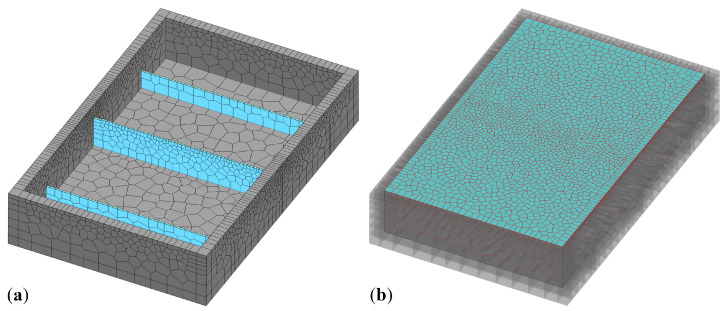
Discretization of test specimens: (**a**) plywood formwork with restraining device inserts and (**b**) concrete specimen cast within the formwork.

A VCLM discretization of the test specimens and plywood formwork is shown in Figure 14. The study of reinforcing bars within the concrete volume requires three-dimensional simulations. The thermal–hygral–mechanical model employed herein utilizes the analysis framework developed by Pan et al. [49], which has the following features:

- Cementitious materials hydration is based on the model proposed by Riding et al. [50]. The degree of cementitious materials reaction can be expressed by the amount of chemically combined water, or equivalently by
  (1) $$\alpha \left(t\right)=\frac{H(t)}{H_{u}}$$
  where H(t) is the cumulative amount of heat produced by the hydration reaction (J/g) and $H_{u}$ is total heat available for reaction (J/g). The total available heat depends on the composition and proportioning of the cementitious materials [50].
- The temperature field within the model domain is simulated using a lattice composed of conduit elements. A convective boundary layer models the heat transfer between the exposed surface of the specimen and the environment. The convective heat exchange $q_{c}$ across the exposed surfaces (including the outer surfaces of the plywood formwork) depends on the difference between the solid surface temperature $T_{s}$ and that of the surrounding ambient medium $T_{a}$
  (2) $$q_{c}=\Lambda_{T}\left(T_{s}-T_{a}\right)$$
  where $\Lambda_{T}$ is the coefficient of convective heat transfer, which can be modeled as follows [51]:
  (3) $$\Lambda_{T}=\left\{\begin{array}{cc} 5.6+4.0v & \mathrm{for}\;v\leq 5\;m/s\\ 7.2v^{0.78} & \mathrm{for}\;v>5\;m/s \end{array}\right.$$
  where *v* is wind speed over the concrete surface. The heat of hydration is calculated at the lattice nodes and used as a source term in the thermal analyses.
- The humidity field is simulated using a parallel set of conduit elements based on the same set of nodal points and element connectivities. After the concrete sets, moisture transport is assumed to be governed by a diffusion process (i.e., according to Fick’s second law). A convective boundary layer accounts for moisture transfer between the drying surface of the concrete and the environment. The modeling of this layer is analogous to that of the temperature field expressed by Equation (2), although it is governed by different mechanisms.
- The solution process involves a time-stepping scheme in which the temperature and humidity fields are solved for each time step using the Crank–Nicholson method in conjunction with a fixed-point algorithm to achieve convergence.
- The simulated thermal and hygral fields produce thermal and hygral strains, respectively, within the mechanical component of the analysis framework, which is based on the rigid body–spring concept [52]. These strain increments produce stress that might lead to crack formation.
- Concrete property development, including strength and stiffness, depends on the degree of cementitious materials hydration. In particular, solidification theory is used to simulate creep processes and stiffness development [53,54]. The development of tensile strength is represented by
  (4) $$f\left(\alpha \right)=f\left(\alpha_{u}\right){\left(\frac{\alpha -\alpha_{0}}{\alpha_{u}-\alpha_{0}}\right)}^{\zeta }\;\;\;\mathrm{for}\;\;\;\alpha \geq \alpha_{0}$$
  where $f(\alpha_{u})$ is the tensile strength at the ultimate degree of hydration, $\alpha_{0}$ is the degree of hydration associated with setting, and the coefficient ζ = 1 when the tensile strength is being modeled [55].
- At a given stage of strength development, concrete fracture is simulated using an energy-conserving crack band model [56,57]. The geometry of neighboring Voronoi cells define the extents of the crack band.
- Each reinforcing bar is represented by series of frame elements, which connect to the concrete nodes via special link elements [58]. The link elements account for the nonlinear bond actions between the concrete and reinforcement, including FRP bars, as described by Focacci et al. [59].

**Figure 15:**
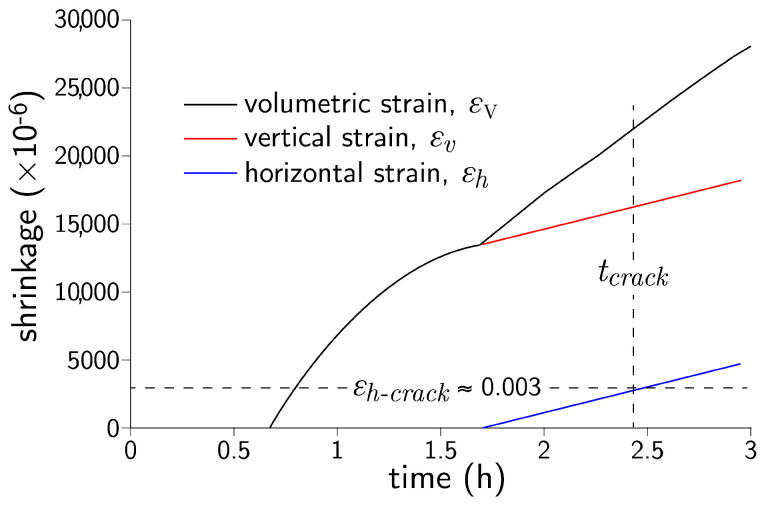
Volumetric instability of concrete in the plastic state (adapted from Ghourchian et al. [60]).

The above model was modified to approximate behavior of concrete in the plastic state. In particular, the modeling of plastic shrinkage was based on the simulations of Ghourchian et al. [60], as presented in Figure 15. Plastic settlement produces volumetric strain that begins with a vertical component. A smaller horizontal strain component appears at a later time. By studying the behavior of different concrete types, Ghourchian et al. [60] suggested that crack initiation is governed by a limiting value of the horizontal strain (e.g., $\epsilon_{h-crack}\;\approx \;0.003$) rather than by a stress measure.

Herein, the volumetric strain is introduced into the lattice model from the time of horizontal strain commencement, while the preceding accumulation of vertical strain is neglected. This volumetric strain continues until the end of the plastic stage, after which drying shrinkage is assumed to be caused by moisture diffusion, as described above. The rate and duration of this form of volumetric strain are model parameters. The stress-based fracture criterion, as expressed by Equation (4), is retained.

The model was further extended to include the effects of evaporation on concrete temperature. Heat flux due to evaporative cooling is determined by

(5) $$q_{e}=-E_{c}\;h_{l}$$

where $E_{c}$ is evaporation rate from the concrete surface (kg/m${}^{2}$/h) and $h_{l}$ is latent heat of vaporization (J/kg). The evaporation rate from the fresh concrete surface is calculated according to Uno [5] in the following form:

(6) $$E_{c}^{\prime }=5\left(V+4\right)\left[{\left(T_{c}+18\right)}^{2.5}-r{\left(T_{a}+18\right)}^{2.5}\right]\times 10^{-6}$$

where *V* is wind velocity (km/h), $T_{c}$ is the temperature of the concrete surface (°C), $T_{a}$ is the ambient temperature (°C), and *r* is the relative humidity of the environment. The evaporation rate is modified to account for maturation of the concrete according to

(7) $$E_{c}=E_{c}^{\prime }\;\exp\left[-{\left(t/a\right)}^{1.5}\right]$$

where *t* is the maturation time (h) and *a* is a time constant set to 3.75 h for concrete [61].

## 6. Simulation Results and Discussion

### 6.1. Thermal Analyses

As a first step, the degrees of hydration at the initial and final setting points as determined through the aforementioned penetration tests were further calibrated. For this purpose, the 150 mm × 150 mm × 150 mm samples were modeled using the VCLM while accounting for the environmental boundary conditions. The coarse aggregate fraction of the mixture composition (Table 2) was removed through the sieving process, thereby increasing the cement content to 687 kg/m${}^{3}$. By plotting the resulting curve for degree of hydration alongside the penetration resistance data (Figure 13), the degree of hydration at initial setting was found to be $\alpha_{0}$ = 0.040. This value is in general agreement with experimentally measured values for the initial setting of cement pastes [62]. The degree of hydration at final setting was $\alpha_{f}$ = 0.089.

For the simulations of temperature development in the slab specimens, the coefficient of convective heat transfer (Equation (3)) was multiplied by a factor of 2/3. Otherwise, all input quantities were set according to the mixture design (Table 2), the experimental measurements (e.g., the recorded wind speed, ambient temperature, and relative humidity), or expressions reported in the literature as outlined in Section 5. Specimen temperature was measured using a thermocouple device inserted through the plywood formwork and extending into the concrete specimen. A computational node was introduced at the location of the thermocouple tip in order to have one-to-one correspondence with respect to the measurement location.

**Figure 16:**
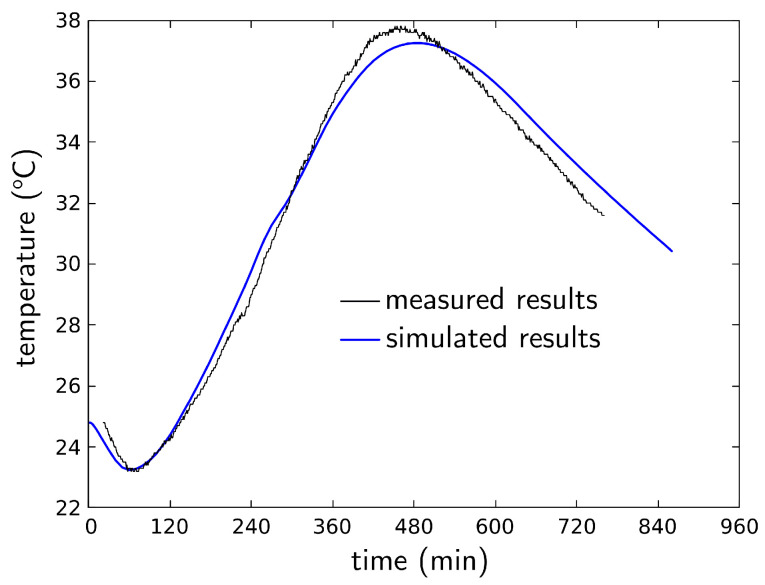
Physically measured and numerically simulated temperatures at the thermocouple location in specimen S-SC-1.

**Figure 17:**
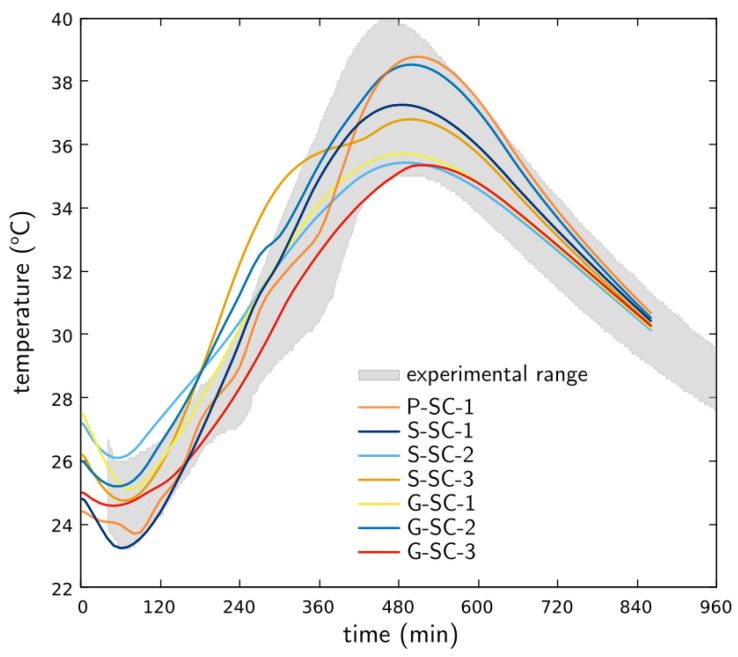
Numerically simulated temperatures for selected slab specimens. The experimentally measured range of temperature values is presented for comparison.

Figure 16 compares the measured and simulated temperature results from the time of casting for specimen S-SC-1. A fuller set of comparisons is provided in Figure 17, where the simulation results for each specimen are plotted on top of the range of measured temperature values. The experimental range does not account for specimens P-SC-2 and P-SC-3, which were deemed to be outliers in terms of their low temperature readings. Whereas the simulation results for S-SC-1 were best in terms of quantitative agreement with the measured results, the set of simulated curves follows the experimentally observed trends reasonably well. Each of the numerical specimens is identical with respect to the model definition, such that the differences in temperature seen in Figure 17 are solely attributable to the environmental exposure conditions along with the initial temperature of the concrete. The initial decline in temperature was typical of the results of most of the specimens. In the numerical simulations, the decline was found to be caused mainly by evaporative cooling, rather than other environmental factors.

### 6.2. Cracking Behavior of Unreinforced Slab Specimens

To simulate the cracking behavior of the slab specimens, the preceding thermal analyses were coupled with hygral and mechanical analysis modules [49]. The contact planes between the concrete and formwork were assumed to be frictionless, in accordance with the steps taken within the experimental program. For the preceding analyses, the recorded ambient temperature, relative humidity, and wind speed were used as environmental boundary conditions.

Prior to simulating plastic shrinkage in terms of volumetric strain, as shown in Figure 15, attempts were made to simulate plastic shrinkage as a diffusion phenomenon, as was done for drying shrinkage. In addition to being conceptually invalid, these attempts failed in several respects. The resulting crack widths at *t* = 6 h were much smaller than those observed in the experiments. Furthermore, significant distributed microcracking occurred over the drying surface, and the resulting macrocrack (which formed over the central device) propagated from the upper surface downward. In contrast, by introducing plastic shrinkage as a volumetric strain, the central device acts as a stress (or strain) riser such that cracking initiates at the device tip and propagates upward. Ghourchian et al. [60] observed such upward propagation of plastic shrinkage cracking from images produced by X-Ray radiography.

The range of simulations covered one representative case for each reinforcement configuration, including simulations of P-SC-1, S-SC-1, and G-SC-1. For concrete in the fresh state, finite amounts of stiffness and strength are needed to operate the mechanical model and represent the cohesive behavior of the concrete mixture. In the absence of such data, the initial stiffness and tensile strength were set at $0.002\;E(\alpha_{u})$ and $0.02\;f(\alpha_{u})$, respectively, in order to achieve cracking behavior comparable to that observed in the test specimens. Because cracking occurs prior to setting of the concrete, the assignment of these values is particularly consequential.

**Figure 18:**
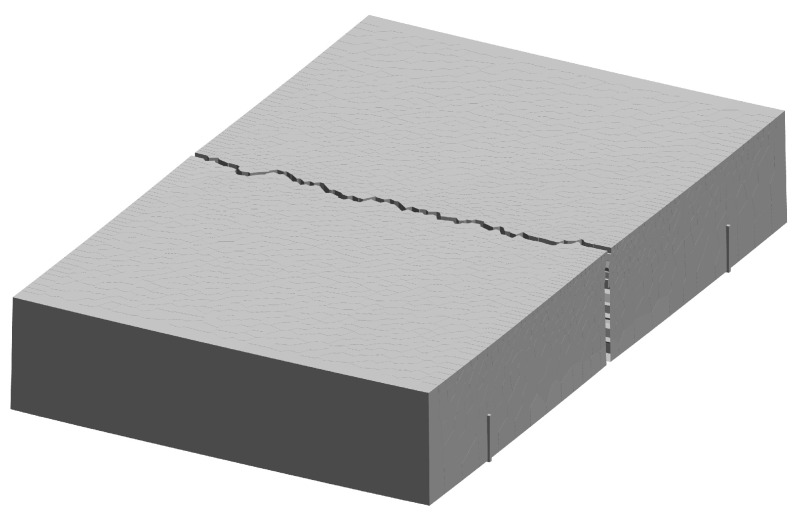
Numerically simulated crack pattern in specimen P-SC-1 at *t* = 6 h.

For the case of P-SC-1, which served as a control specimen without reinforcement, cracking in the model is initiated at *t* = 130 min at the tip of the central restraining device. The crack propagates upward and becomes visible on the drying surface at *t* = 170 min. The time of travel of the crack from the device tip to the concrete surface is in general agreement with radiographic image-based observations [60]. Rapid crack growth occurs during the period of horizontal straining indicated in Figure 15. In accordance with the experimental results, cracks form across the midspan width of the specimen over the central riser (Figure 18). The discontinuous cracking behavior observed in several of the laboratory specimens (Figure 5) was not seen in the simulations, possibly due to lack of heterogeneity in the material model. Although not evident in Figure 18, local microcracking occurs at the peripheral restraints, which has been observed in other experimental programs as well [8].

**Figure 19:**
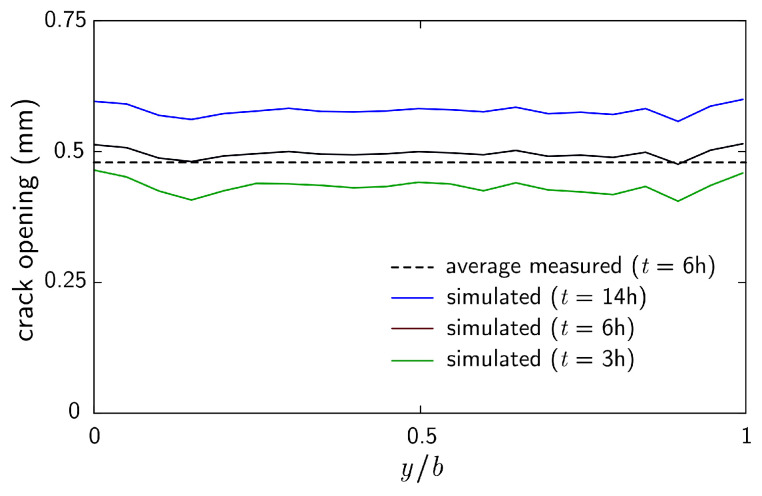
Numerically simulated crack opening profiles for specimen P-SC-1 (where *y* represents the position along the crack within width *b* of the specimen). The experimentally measured average crack opening is presented for comparison.

Crack opening profiles are presented in Figure 19 for several time durations. Herein, the rate of horizontal straining was set at 1800 μ/h, which is approximately half that indicated in Figure 15. This lower rate of volumetric straining of the bulk material led to roughly the same average crack opening (0.48 mm at *t* = 6 h) as that observed in the experimental program. Beyond the time of concrete setting, drying shrinkage contributes to additional crack opening. From about *t* = 8 h onward, cooling of the concrete causes contraction and additional crack opening. Over this short time span, however, the contributions of drying shrinkage and thermal contraction to crack opening are relatively small.

For all of the slab simulations, the initial driver of crack formation and development (i.e., volumetric plastic strain) was introduced as a prescribed function of time independent of concrete maturity and the environmental boundary conditions. Furthermore, the influences of plastic settlement were not included in this modeling effort. Lack of cracking observed along the reinforcing bar suggests that plastic settlement is not a primary factor in initiating cracking, though further investigation is needed. In Pan et al. [63], the VCLM was extended to simulate such hygromechanical behavior using the effective stress concept of Biot [64]. Transport of water in porous media is driven by gravity and described using Darcy’s Law in rate format. Work towards integrating this capability with fracture modeling and the other relevant chemophysical processes is ongoing.

### 6.3. Cracking Behavior of Reinforced Slab Specimens

As noted in Section 5, each reinforcing bar is represented by a series of frame elements which connect to concrete nodes via nonlinear link elements. The frame elements are assigned the geometric and material properties of the steel or GFRP bars, as the case may be. The bond properties assigned to the link elements were determined through the aforementioned pullout tests conducted at *t* = 2 h, the approximate time of plastic shrinkage crack initiation. More specifically, bonding between the reinforcement and concrete was modeled using piecewise linear fittings of the *t* = 2 h test results presented in Figure 10.

**Figure 20:**
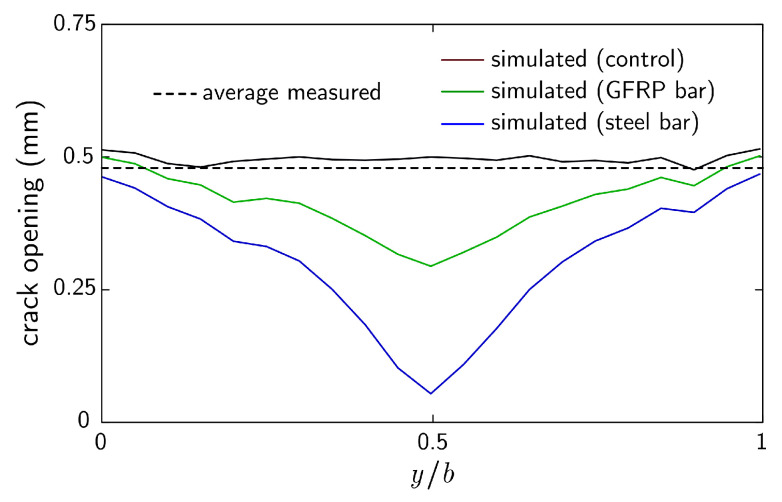
Numerically simulated crack opening profiles for specimens with and without reinforcement at *t* = 6 h. The experimentally measured average crack opening at *t* = 6 h is presented for comparison.

Figure 20 presents the simulated crack opening profiles at *t* = 6 h for specimens S-SC-1 and G-SC-1, which contained steel bar and GFRP bar reinforcement, respectively. For comparison, the opening profile for specimen P-SC-1 is presented along with the average crack opening measured for all specimens within the experimental program. The presence of bar reinforcement does not prevent crack formation, though the simulated crack openings are locally restricted to the bar position. Considering the low bond strength for the GFRP bar at *t* = 2 h, its ability to restrict crack opening might be surprising. However, the concrete material is relatively compliant over the same time period, such that even small bond strength enables significant load transfer between the reinforcement and concrete. Short distributed fibers, which supply a much larger bonded surface area, are a common means of avoiding this form of plastic shrinkage cracking.

## 7. Conclusions

In this research, physical testing and numerical modeling have been used to study the performance of GFRP bars in restraining early-age cracking in concrete slabs. The period of interest begins with the concrete in a fresh state and extends up to several hours after setting. For comparison, concrete slabs reinforced with steel bars were examined, as well as control specimens without reinforcement. Several conclusions can be made.

- Consistent cracking behavior of the control specimens was obtained by optimizing the concrete mixture design, controlling the environmental boundary conditions, and raising the initial temperature of the concrete, which increased the evaporation rate from the drying surface. The initial temperature was raised by heating the mixing water before its addition to the dry materials. Cracking initiated with the concrete in a plastic state prior to setting.
- For the test specimens considered herein, crack opening does not appear to be restricted in the vicinity of a bar; rather, openings are fairly uniform over the specimen width. In other words, the presence of reinforcing bars of either type, i.e., GFRP or steel, does not have an appreciable effect on time of plastic shrinkage crack initiation and ensuing crack opening.
- Bond strength between both types of reinforcement and the early-age concrete was measured and found to be small (e.g., the maximum bond strength at *t* = 2 h was only 0.14 MPa and 2.08 MPa for GFRP and steel bars, respectively). Such low bond strength might be responsible for the ineffectiveness of the reinforcement in restraining plastic shrinkage cracking.
- It was observed that the plastic shrinkage cracking can occur for evaporation rates that are even below the commonly accepted critical value of 1.0 kg/m${}^{2}$/hr (0.2 lb/ft${}^{2}$/hr).
- The proposed numerical analyses elucidate the roles of the various thermo-hygro-mechanical processes that affect early-age cracking of the specimens. In particular, by modeling plastic shrinkage as a bulk material phenomenon in the form of volumetric strain, the central device acts as a stress riser. The simulated cracking initiates from the device tip and propagates upward, as seen in other studies using images based on X-Ray radiography.
- Concrete temperatures, and as such the properties of the concrete, are influenced by the thermal properties of the formwork and related boundary conditions. The modeling of evaporative cooling was found to be essential in simulating the concrete temperatures, particularly early on in the testing period, where measured temperature tended to decrease with time.
- Our numerical analyses support the notion that discrete bar reinforcement does not prevent the formation of plastic shrinkage cracks. In contrast to the experimental observations, however, the presence of the bar reinforcement does restrict crack opening. Higher bond strength leads to smaller crack openings local to the bar. The differences between the measured and simulated opening profiles are grounds for further study.

Although the modeling exercises provide insight into the use of GFRP bars to reduce plastic shrinkage cracking, a number of simplifying assumptions have been made. In particular, volumetric strain due to plastic settlement/shrinkage was introduced as a prescribed function of time, patterned after experimental observations in previous studies. The use of a strength-based fracture model required additional assumptions the regarding baseline values of strength and stiffness representing the cohesive behavior of the fresh concrete. Further development of the model with respect to these points is needed.

For practical situations in which plastic shrinkage cracking is a concern, the results presented herein indicate that routinely-spaced GFRP bar reinforcement is not effective in preventing crack formation and development. Due to their larger specific surface area, well-distributed short fibers are a proven means of addressing the problem of plastic shrinkage cracking.

## Figures and Tables

**Table 1:** Cement composition.

Item	wt%
CaO	64.1
SiO${}_{2}$	20.3
Al${}_{2}$O${}_{3}$	4.8
Fe${}_{2}$O${}_{3}$	3.5
MgO	0.9
SO${}_{3}$	2.7
Na${}_{2}$O	0.12
K${}_{2}$O	0.30
Loss on ignition	2.5

**Table 2:** Concrete mixture design.

Constituent	kg/m${}^{3}$	lb/yd${}^{3}$
Portland Cement Type I/II	517	871
Water	291	491
Coarse Aggregate # 89	669	1128
Fine Aggregate (Sand)	860	1449
Unit Weight	2337	3939
*w*/*c*	0.56	

**Table 3:** Physical and geometrical properties of GFRP rebar.

GFRP Rebar	Bar Designation No.	Fiber Type	Nominal Diameter, mm [in]	Nominal Cross-Sectional Area, mm${}^{2}$ [in${}^{2}$]	Measured Cross-Sectional Area, mm${}^{2}$ [in${}^{2}$]	Fiber Content, %
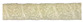	M10 [3]	Glass	9.5 [0.375]	71 [0.11]	87.67 [0.136]	80

**Table 4:** Mechanical properties of GFRP rebar.

Guaranteed Tensile Strength, MPa [ksi]	Modulus of Elasticity, GPa [Msi]	Mean Ultimate Tensile Strain	Guaranteed Bond Strength, MPa [ksi]
952 [138.1]	60.5 [8.78]	1.9	1.84 [0.27]

**Table 5:** Measured crack data at six hours after concrete casting.

Specimen ID	Reinforcement	Crack Onset, min	Crack Area, mm${}^{2}$ [in${}^{2}$]	Crack Length, mm [in]	Crack Width, mm [in]
P-SC-1	Plain	139	133.5 [0.207]	345.4 [13.6]	0.39 [0.015]
P-SC-2	Plain	132	137.4 [0.213]	290.0 [11.4]	0.47 [0.019]
P-SC-3	Plain	131	202.6 [0.314]	375.8 [14.8]	0.54 [0.021]
S-SC-1	Steel	113	246.5 [0.382]	353.1 [13.9]	0.70 [0.027]
S-SC-2	Steel	141	73.5 [0.114]	188.0 [7.4]	0.39 [0.015]
S-SC-3	Steel	131	129.7 [0.201]	342.9 [13.5]	0.38 [0.015]
G-SC-1	GFRP	115	112.9 [0.175]	340.4 [13.4]	0.33 [0.013]
G-SC-2	GFRP	NA	58.1 [0.090]	139.7 [5.5]	0.42 [0.016]
G-SC-3	GFRP	120	219.4 [0.340]	322.6 [12.7]	0.68 [0.027]

**Table 6:** Penetration resistance and time of setting results.

Specimen ID	Elapsed Time, min	Penetration Resistance, MPa [psi]	Set Type	ASTM Set Time, min	Fit Curve Set Time, min
	171	1.4 [204]			
	214	6.9 [1000]	Initial set (IS)	196	
1	259	19.3 [2800]			
	287	40.3 [5840]	Final set (FS)	280	
	323	57.4 [8320]			
	175	1.4 [204]			
	215	7.2 [1040]	Initial set (IS)	194	IS = 203
2	258	18.5 [2680]			
	286	37 [5360]	Final set (FS)	292	FS = 270
	323	48.3 [7000]			
	177	1.9 [272]			
	216	7.2 [1040]	Initial set (IS)	187	
3	256	20.4 [2960]			
	285	35.9 [5200]	Final set (FS)	288	
	322	56.8 [8240]			

## Abbreviations

The following abbreviations are used in this manuscript:

ASTM	American Society for Testing and Materials
ACI	American Concrete Institute
FRP	Fiber-Reinforced Polymer
GFRP	Glass Fiber-Reinforced Polymer
LVDT	Linear Variable Differential Transformer
UTM	Universal Testing Machine
VCLM	Voronoi Cell–Lattice Model
